# Mild hyperbaric hyperoxia improves aerobic capacity and suppresses cardiopulmonary stress during the maximal cycle-ergometer test

**DOI:** 10.1371/journal.pone.0323885

**Published:** 2025-05-23

**Authors:** Kazufumi Hisamoto, Naoki Okubo, Mako Fujita, Hideki Fukushima, Yoshinori Okizuka, Takashi Yamanaka, Tomoyuki Matsui, Toru Morihara, Tatsuya Hojo, Yoshiyuki Fukuoka, Kenji Takahashi

**Affiliations:** 1 Department of Orthopaedics, Graduate School of Medical Science, Kyoto Prefectural University of Medicine, Kyoto, Japan; 2 Faculty of Health and Sports Science, Doshisha University, Kyoto, Japan,; 3 Marutamachi Rehabilitation Clinic, Kyoto, Japan; Amazonas State University, BRAZIL

## Abstract

Aerobic exercise is more recommended than anaerobic exercise for individuals with cardiopulmonary dysfunction to avoid cardiopulmonary stress. However, their oxygen-carrying capacity is already reduced, making it difficult to exercise enough. Therefore, we aimed to investigate whether mild hyperbaric hyperoxia enhances aerobic capacity and decreases cardiopulmonary stress during exercise with a particular focus on the ventilatory threshold (VT). Nineteen healthy men (age 34.9 ± 10.8 years) performed ramp-loading tests on a cycle-ergometer under the three oxygen conditions: hypobaric hypoxia (HYPO; 0.7 ATA, 14.7% O_2_), normobaric normoxia (NOR; 1.0 ATA, 21% O_2_), and mild hyperbaric hyperoxia (HYPER; 1.3 ATA, 35% O_2_). Cardiopulmonary data were recorded using a gas exchange analyzer. VT was assessed based on minute ventilation (V_E_) using the V-slope method and the work rate on VT (W_VT_) was determined. Although the maximal values of V_E_ did not differ among the oxygen conditions, W_VT_ in the HYPER condition was significantly higher compared to others (HYPO; 125 ± 21, NOR; 148 ± 24, HYPER; 168 ± 32 [W], each p < 0.01). Systolic blood pressure and double product on VT in the HYPER condition were significantly reduced compared to others (HYPO; 172 ± 21, 23096 ± 4354, NOR; 173 ± 15, 23377 ± 3109, HYPER; 155 ± 18, 21255 ± 3340 [mmHg, beats·min^-1^·mmHg], each p < 0.05). Although further clinical research targeting other populations is needed to apply mild hyperbaric hyperoxia in clinical practice, due to its positive effects on W_VT_ and cardiopulmonary stress, the HYPER oxygen condition may potentially be used to enhance aerobic capacity and make individuals with cardiopulmonary dysfunction exercise safely.

## Introduction

With the rapid aging of the population worldwide, an increasing number of elderly people have cardiopulmonary dysfunction and declined exercise tolerance [1–3]. In clinical practice, aerobic exercise is recommended for elderly patients with cardiopulmonary dysfunction [4–6], because cardiopulmonary stress is promoted under anaerobic metabolism [7]. The ventilatory threshold (VT) is defined as the turning point from aerobic to anaerobic metabolism [8]. Below the VT, sufficient oxygen is supplied to the skeletal working muscles, and the matching of O_2_ supply and O_2_ utilization is maintained. We attempted to increase the O_2_ supply during exercise by raising atmospheric pressure and oxygen concentration.

Hyperbaric oxygen therapy is used for the treatment of various diseases such as carbon monoxide intoxication and decompression sickness and is generally set to 2.0 or more ATA (atmospheres absolute) and 100% O_2_ concentrations. Barotrauma [9,10], and restriction of pulmonary function [11] have occasionally been observed as adverse events related to this hyperbaric oxygen therapy. For the clinical rehabilitation of the elderly, adverse events due to excessive pressure and O_2_ levels need to be avoided. Therefore, we focused on the oxygen condition (1.3 ATA, 35% O_2_), with a slight increase in atmospheric pressure and oxygen concentration. Oxygen conditions of 1.25–1.3 ATA with 35–40% O_2_ have been defined as mild hyperbaric hyperoxia [12]. A previous study reported that exposure to this oxygen condition stimulated the parasympathetic nervous system in healthy individuals [13]. Parasympathetic excitation is expected to suppress hypertension and tachycardia, reducing cardiopulmonary stress. In another study, rat models of hypertension exposed to mild hyperbaric oxygen showed lower blood pressure than those kept under normoxia [14].

Parasympathetic excitation may help suppress an increase in blood pressure and cardiopulmonary stress, enabling safer exercises for individuals with cardiopulmonary dysfunction. We hypothesized that mild hyperbaric hyperoxia could help maintain aerobic metabolism during exercise without increasing cardiopulmonary stress.

However, given the lack of previous research on exercise under mild hyperbaric hyperoxia, it was essential first to evaluate its effects on cardiopulmonary function and safety in healthy individuals. Therefore, to clarify our hypothesis, this study investigated changes in aerobic metabolism and cardiopulmonary stress during maximal exercise in healthy participants.

## Methods

### Participants

Nineteen healthy young and middle-aged male participants were enrolled in this study (age 34.9 ± 10.8 years, height 172 ± 5 cm, weight 68.7 ± 5.9 kg, mean ± standard deviation [SD]). They were medical professionals, including physical trainers and doctors. None of the participants had a history of chronic cardiopulmonary dysfunction.

After approval from the Ethics Committee of Marutamachi Hospital (IRB approval number: 01–00021), participants were recruited, and informed written consent was obtained from all participants. Participants were recruited from November 1, 2021 to April 30, 2022. This study was conducted in accordance with the ethical principles outlined in the Declaration of Helsinki for research involving human subjects.

### Oxygen environments

The exercise tests were performed in customized barometric pressure-controlled chambers (2WAY-DX Free Space; W2500 × D4000 × H2600 mm, Japan Press Bulk Ind., Shizuoka, Japan). The chamber was maintained at 25ºC with 50% relative humidity. Three oxygen conditions were set: hypobaric hypoxia (HYPO; 0.7 ATA, 14.7% O_2_), normobaric normoxia (NOR; 1.0 ATA, 21% O_2_), and mild hyperbaric hyperoxia (HYPER; 1.3 ATA, 35% O_2_). The condition of 0.7 ATA is equivalent to an altitude of 3,000 m [15]. The HYPER condition was achieved by adjusting the rate of O_2_ volume with an oxygen concentrator (ZYJ-10, Nanjing Careland Medical Equipment Co., Nanjing, China).

### Maximal exercise protocol

The participants performed exercise load tests using a cycle ergometer (75XL-III; Konami, Tokyo, Japan) on 3 different days, with a 1-week interval between each test. The order of the three oxygen conditions was determined randomly and was not disclosed to the participants. The maximal exercise load test was performed on the cycle ergometer until the participant’s exhaustion was observed. The gas analyzer mask was fitted to the participant’s nose and mouth to ensure that there was no leakage from the sides of the mask.

**Fig 1:**
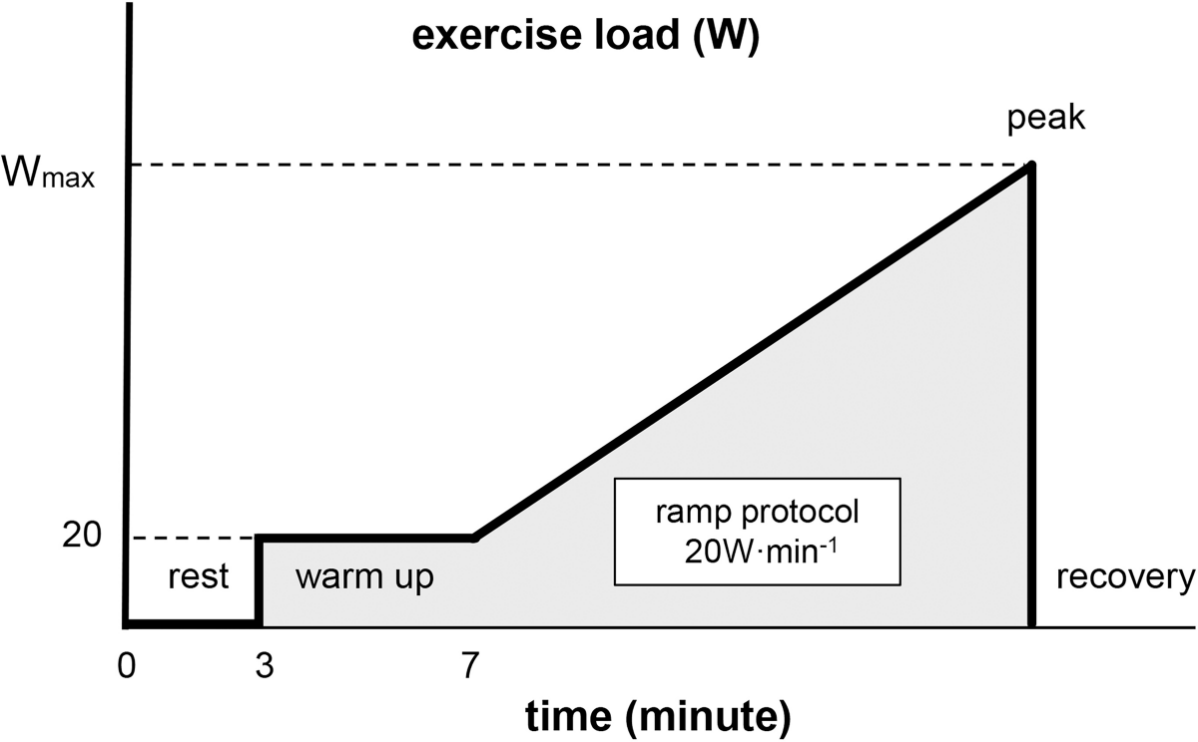
The protocol of the maximal cycle-ergometer test.

The participants performed the exercise test on a cycle ergometer after three minutes of resting on the saddle (rest phase). The exercise load began at 20 watts (W) for four minutes (warm-up phase) and was increased by 20 W every minute until exhaustion was observed (ramp-protocol phase). The endpoint of the ramp protocol period was defined as the peak point. The peak work rate was defined as the maximum work rate (W_max_). After the peak point, the participants continued pedaling with unloading for one minute (recovery phase). The participants were encouraged to maintain a cadence of 60 rpm with the assistance of a metronome throughout the maximal cycle ergometer test (Fig 1).

### Measurements

#### Pulmonary gas exchange response.

During the exercise test, pulmonary gas exchange variables were continuously monitored using a computerized online breath-by-breath measuring system (AE-310s; Minato Medical Sciences, Osaka, Japan). The metabolic chart was calibrated before each exercise test according to the manufacturer’s recommendations. The breath-by-breath data were averaged every ten seconds.

The flow sensor of the AE-310s was a hot-wire velocimeter, and calibration was performed based on temperature, humidity, and atmospheric pressure. Flow meter calibration accuracy for the AE-310s was guaranteed under HYPO, NOR, and HYPER conditions. Therefore, minute ventilation (V_E_), tidal volume (TV), and breath frequency (BF) were measured under the three oxygen conditions. In contrast, gas meter calibration accuracy for the AE-310s was guaranteed only under 0.7–1.05 ATA. Thus, the following measurements were taken only under the NOR condition: oxygen uptake (VO_2_), carbon dioxide elimination (VCO_2_), end-tidal O_2_ pressure (P_ET_O_2_), and end-tidal CO_2_ pressure (P_ET_CO_2_). Ventilatory equivalents of oxygen (V_E_/VO_2_), carbon dioxide (V_E_/VCO_2_), and oxygen pulse (VO_2_/HR) were calculated. Respiratory gas exchange ratio (R) was estimated by the equation “R=VCO_2_/VO_2_”, and used to evaluate whether maximal exercise was performed [16]. VO_2max_ was automatically calculated as the maximum VO_2_ value per body weight. V_E_/VCO_2_ slope was also automatically calculated.

#### Cardiopulmonary response.

The heart rate (HR) and percutaneous O_2_ saturation (SpO_2_) were recorded every minute by attaching a finger pulse oximeter (Deluxe SM-110; Santa Medical, CA, USA) to the participant’s left second fingertip. Systolic and diastolic blood pressures (SBP and DBP) were recorded every minute using a manual Manchette sphygmomanometer (DS-45; Welch Allyn Japan, Tokyo, Japan) throughout the exercise test. As an index of cardiopulmonary stress, the double product (DP) was evaluated using the formula “DP = HR × SBP” [17]. The work rate relative to W_max_ (%WR) was used to analyze the changes in cardiopulmonary responses during the exercise tests.

#### The criteria for termination.

In addition to the inability to maintain cadence, when a plateau or drop in VO_2_, HR > 95% of the age-predicted maximum (220-age) [18], and severe hypoxemia (SpO_2_ < 80%) were observed, exercise tests were terminated.

#### The method of detecting VT.

**Fig 2:**
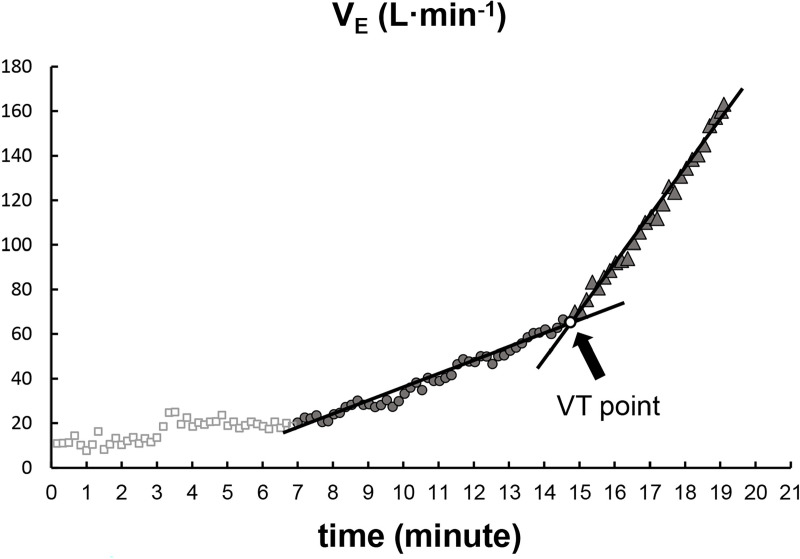
Determination of the ventilatory threshold (VT) point. The examiners determined two regression lines in the early and late phases of V_E_ change. The inflection point of regression lines was defined as the VT point. Abbreviations: V_E_, minute ventilation; W_max_, maximal work rate.

A graph of the change in V_E_ during the ramp protocol period (x-axis: time; y-axis: V_E_) was used to determine VT [19,20]. Three examiners familiar with respiratory gas analysis determined two regression lines in the early and late phases of V_E_ change. The intersection of the two regression lines was defined as the VT point (V-slope method) (Fig 2) [21]. The difference in the work rate on VT (W_VT_) among the three oxygen conditions was evaluated.

### Statistical analysis

All data were presented as means ± SD. Statistical significance was set at p < 0.05. Statistical analyses were performed using the SPSS 26.0 for Windows (IBM Corp., Armonk, NY, USA). One-way repeated analysis of variance (ANOVA) with Bonferroni’s *post-hoc* test was used to identify differences among the three oxygen conditions. The changes in HR, SBP, DP, and SpO_2_ were compared between the oxygen conditions (HYPO, NOR, and HYPER) and %WR (0–100%) using repeated two-way ANOVA. Bonferroni’s *post-hoc* test was applied to appropriate datasets if a significant *F*-value was obtained. We used a sample size determination program (PS: Power and Sample Size Calculation ver. 3.1.6 by D.D. William and D.P. Walton) to determine whether the sample size was adequate to detect differences among FIO_2_ conditions. This study was designed to analyze a continuous response variable from matched pairs of participants. Since no studies have investigated exercise under hyperbaric hyperoxic conditions, we referred to a previous study on exercise under hypoxic conditions. In our previous study [22], the difference in the ventilatory threshold of matched pairs within two conditions (normoxia and 12% hypoxia) was normally distributed with an SD of 21.2 (W). If the true difference in the mean response of matched pairs was 49.0 (W), it was necessary to study at least 5 participants to reject the null hypothesis that this response difference was zero with the probability (power) value of 0.8. The probability of Type I error associated with this test of the null hypothesis was 0.01.

## Results

### Validity of maximal exercise test under NOR condition

The cardiopulmonary variables at rest and peak under the NOR condition are summarized in Table 1. At rest, HR was 70 ± 9 beats·min^-1^, SBP was 120 ± 10 mmHg, and SpO_2_ was 98 ± 1%. At peak, ramp-exercise time was 603 ± 122 seconds, HR was 169 ± 11 beats·min^-1^, SBP was 205 ± 23 mmHg, and SpO_2_ was 97 ± 2%. R at peak was 1.11 ± 0.06, indicating that all participants had performed the maximal exercise test [23]. VO_2max_ was 43.9 ± 9.3 mL·min^-1^·kg^-1^.

**Table 1:** Cardiopulmonary variables at rest and peak under the NOR condition.

variables		rest			peak		
V_E_	(L·min^-1^)	10.8	±	2.8	106.3	±	20.4
TV	(mL)	652	±	129	2208	±	328
BF	(breaths·min^-1^)	16.6	±	3.8	49.1	±	11.1
ramp-load time	(second)		–		603	±	122
W_max_	(W)		–		221	±	41
VO_2_	(mL·min^-1^)	319	±	58	2994	±	558
VO_2max_	(mL·min^-1^·kg^-1^)		–		43.9	±	9.3
VCO_2_	(mL·min^-1^)	235	±	46	3311	±	531
R		0.74	±	0.10	1.11	±	0.06
V_E_/VO_2_		34.3	±	9.4	35.7	±	4.1
V_E_/VCO_2_		47.1	±	14.3	32.0	±	3.0
O_2_ pulse	(mL·beats ^-1^)	4.5	±	0.8	15.6	±	1.5
V_E_/VCO_2_ slope			–		25.5	±	1.9
P_ET_O_2_	(mmHg)	15.0	±	1.2	121.5	±	3.4
P_ET_CO_2_	(mmHg)	5.1	±	0.4	40.6	±	3.0
HR	(beats·min^-1^)	70	±	9	169	±	11
SBP	(mmHg)	120	±	10	205	±	23
DBP	(mmHg)	83	±	9	90	±	10
SpO_2_	(%)	98	±	1	97	±	2
DP	(beats·min^-1^·mmHg)	8315	±	1267	34639	±	5660

Values are presented as mean ± standard deviation

### Cardiorespiratory response under the three oxygen conditions

**Fig 3:**
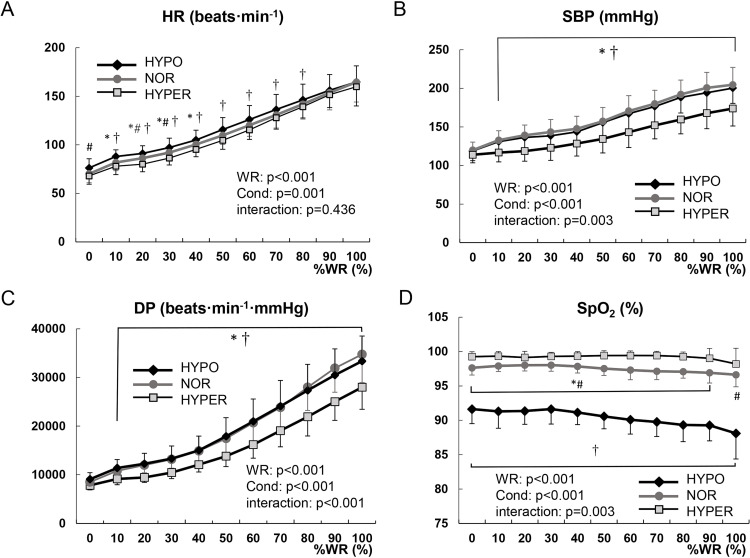
Comparison of the changes in HR (A), SBP (B), DP (C), and SpO2 (D) during maximal exercise test among the three oxygen conditions. Values are mean ± standard deviation. * NOR vs HYPER, # NOR vs HYPO, † HYPER vs HYPO (p < 0.05). The main effect of work rate (WR), the main effect of oxygen condition (Cond), and their interaction were presented. Abbreviations: HR, heart rate; SBP, systolic blood pressure; DP, double product; %WR, work rate relative to maximum work rate.

**Fig 4:**
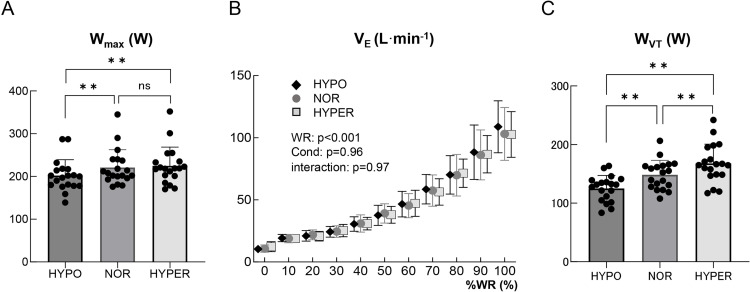
Comparison of W_max_ (A), the changes in V_E_ (B), and W_VT_ (C) among the three oxygen conditions. (A, C) Black dots show the data of each participant. Values are mean ± standard deviation. *p < 0.05, **p < 0.01. (B) Values are mean ± standard deviation. The main effect of work rate (WR), the main effect of oxygen condition (Cond), and their interaction were presented. Abbreviations: W_max_, maximal work rate; V_E_, minute ventilation; W_VT_, work rate on ventilatory threshold; ns, no significant difference; %WR, work rate relative to maximum work rate.

Fig 3 shows the changes in HR, SBP, DP, and SpO_2_ under the different oxygen conditions. HR under the HYPER condition was significantly lower compared to the NOR condition (range; 10–40%WR, p < 0.05) and HYPO condition (range; 10–80%WR, p < 0.05) (Fig 3A). Both SBP and DP were significantly lower under the HYPER condition compared to the other two oxygen conditions (range; 10–100%WR, p < 0.05) (Fig 3B, 3C). The change in SpO_2_ under the HYPER condition was significantly higher compared to the NOR condition (range; 0–90%WR, p < 0.05) and HYPO condition (range; 0–100%WR, p < 0.05) (Fig 3D). There was a significant difference in W_max_ among the three conditions (HYPO; 202 ± 36 W, NOR; 221 ± 41 W, and HYPER; 225 ± 43 W, p < 0.01) (Table 2, Fig 4A). W_max_ under the HYPER condition was significantly higher than that under the HYPO condition (p < 0.01), but not significantly different than that under the NOR condition (p = 0.36). W_max_ under the HYPO condition was significantly lower than that under the NOR condition (p < 0.01). There were no significant differences in V_E_, TV, BF, and HR at peak among the three oxygen conditions. SBP, DBP, and DP at peak under the HYPER condition were significantly lower than those under the other oxygen conditions (p < 0.01). SpO_2_ at peak under the HYPER condition was significantly higher than that under the other oxygen conditions (p < 0.01) (Table 2).

**Table 2:** Cardiopulmonary variables at peak under the HYPO, NOR, and HYPER conditions.

variables		HYPO			NOR			HYPER			p
ramp-load time	(second)	546	±	108	603	±	122	614	±	128	<0.01
W_max_	(W)	202	±	36	221	±	41	225	±	43	<0.01
V_E_	(L·min^-1^)	110.4	±	20.9	106.3	±	20.4	103.1	±	17.9	0.27
TV	(ml)	2140	±	356	2208	±	328	2049	±	352	0.14
BF	(breaths·min^-1^)	52.1	±	8.8	49.1	±	11.1	51.6	±	12.4	0.52
HR	(beats·min^-1^)	168	±	15	169	±	11	169	±	13	0.29
SBP	(mmHg)	200	±	20	205	±	23	174	±	19	<0.01
DBP	(mmHg)	85	±	12	90	±	10	79	±	9	<0.01
SpO_2_	(%)	88	±	4	97	±	2	98	±	2	<0.01
DP	(beats·min^-1^·mmHg)	33298	±	5014	34639	±	5660	27836	±	4444	<0.01

Values are presented as mean ± standard deviation

### VT under the three oxygen conditions

**Fig 5:**
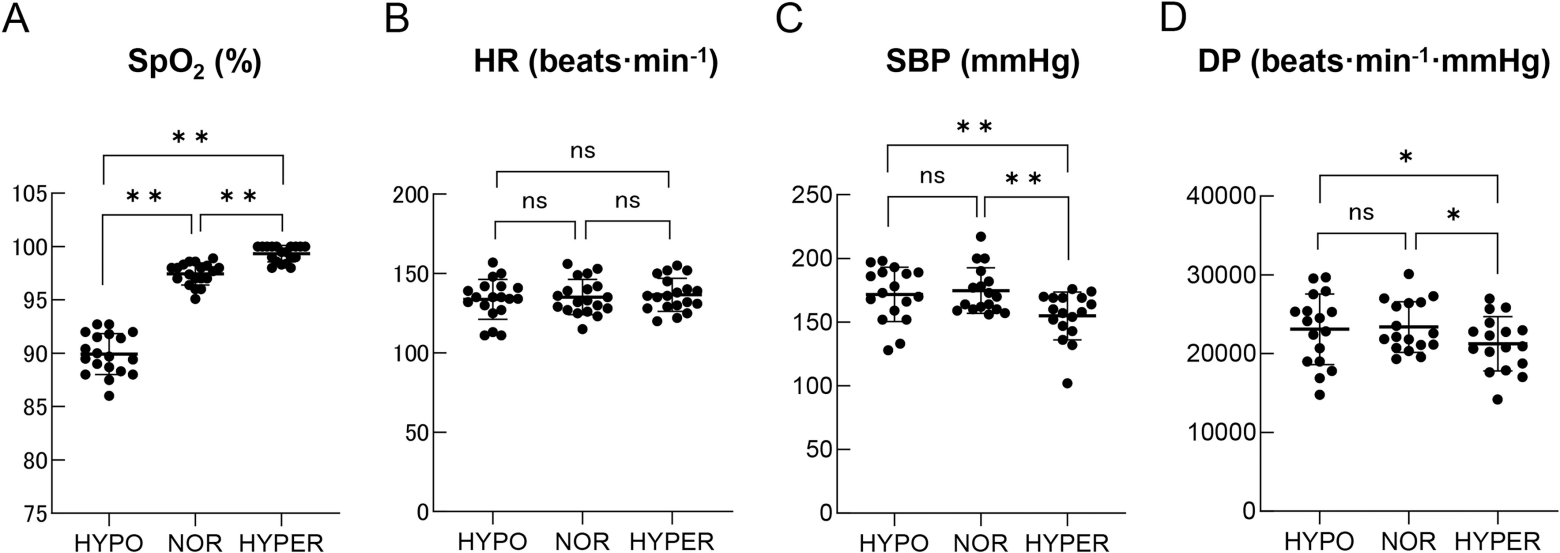
Comparison of SpO_2_ (A), HR (B), SBP (C), and DP (D) on VT among the three oxygen conditions. Black dots show the data of each participant. Values are mean ± standard deviation. *p < 0.05, **p < 0.01. Abbreviations: HR, heart rate; SBP, systolic blood pressure; DP, double product; VT, ventilatory threshold; ns, no significant difference.

Even though there was no significant difference in V_E_ during maximal exercise among the three oxygen conditions with two-way repeated ANOVA (Fig 4B), W_VT_ was significantly higher under the HYPER condition (168 ± 32 W) compared to the HYPO (125 ± 21 W, p < 0.01) and NOR (148 ± 24 W, p < 0.01) conditions (Fig 4C). Regarding cardiorespiratory response on VT, SpO_2_ on VT was significantly higher under the HYPER condition (99.3 ± 0.7%) compared to the HYPO (89.9 ± 1.9%, p < 0.01) and NOR conditions (97.4 ± 1.0%, p < 0.01) (Fig 5A). There was no significant difference in HR on VT among the three oxygen conditions (HYPO; 134 ± 12 beats·min^-1^, NOR; 135 ± 11 beats·min^-1^, HYPER; 137 ± 10 beats·min^-1^) (Fig 5B). SBP on VT was significantly suppressed under the HYPER condition (155 ± 18 mmHg) compared to the HYPO (172 ± 21 mmHg, p < 0.01) and NOR (173 ± 15 mmHg, p < 0.01) conditions (Fig 5C). Moreover, DP on VT under the HYPER condition (21255 ± 3340 beats·min^-1^·mmHg) was significantly suppressed compared to the HYPO (23096 ± 4354 beats·min^-1^·mmHg, p < 0.05) and NOR (23377 ± 3109 beats·min^-1^·mmHg, p < 0.05) conditions (Fig 5D).

## Discussion

In this study, we evaluated the acute effects of the HYPER oxygen condition on aerobic capacity during maximal exercise tests in healthy individuals, with a particular focus on changes in the volume-related parameters and cardiopulmonary response. Although W_max_ did not change under the HYPER condition compared with the NOR condition, W_VT_ was significantly elevated under the HYPER condition compared with the NOR condition. Elevated W_VT_ suggested that the HYPER condition enhanced the participants’ aerobic capacity. Furthermore, the suppression of cardiovascular stress, such as SBP and DP, on VT was observed under the HYPER condition.

The balance between the O_2_ demand and supply in the skeletal muscle affects the determination of VT [24,25]. Even though the O_2_ demand was expected to increase continually during the ramp protocol, W_VT_ under the HYPER condition was higher than that under the other oxygen conditions, indicating that the O_2_ supply to the working muscle was increased under the HYPER condition. The O_2_ supply to working muscle was reported to be determined by arterial oxygen content (CaO_2_) × blood flow [26]. The oxygen in the blood is categorized into Hb-bound O_2_ and non-bound O_2_ (dissolved O_2_). Elevated SpO_2_ levels during the exercise tests under the HYPER condition indicated that Hb-bound O_2_ also increased. The dissolved O_2_ under the HYPER condition was estimated to have increased because dissolved O_2_ is proportional to the O_2_ concentration and atmospheric pressure [26]. Based on the above discussion, under the HYPER condition, both Hb-bound O_2_ and dissolved O_2_ were increased, leading to an increase in CaO_2_. It has been reported that blood flow into working muscles is reduced under normobaric hyperoxia due to vasoconstriction [26]. This was because the expression of vasodilators such as nitric oxide (NO) [27] or prostaglandins (PG) [28] are suppressed under normobaric hyperoxia. Although blood flow was not directly evaluated in this study, hyperbaric hyperoxic conditions were estimated to induce high pressure on the peripheral organs and vessels, resulting in a greater reduction in blood flow than normobaric hyperoxia conditions. Therefore, the increased O_2_ supply under the HYPER condition was mainly affected by an increase in CaO_2_ rather than the change in blood flow. Under the HYPER condition, significant decreases in cardiopulmonary stress indicators were observed. This effect under the HYPER condition was regarded as the most notable benefit for the elderly to exercise more safely. Although the mechanism of the cardiovascular response under the HYPER condition remains unclear, it has been reported that exposure to hyperbaric hyperoxic conditions causes parasympathetic excitation [13,29,30].

Previous studies have demonstrated that exposure to hyperbaric hyperoxia (2.5 ATA, 100% O₂) during rest suppresses an increase in heart rate more effectively than normobaric hyperoxia (1.0 ATA, 100% O₂) [31]. Even with the same oxygen concentration, differences in atmospheric pressure may affect the degree of parasympathetic excitation. Regarding the effects of oxygen conditions during exercise, another study reported that normobaric hyperoxia (1.0 ATA, 60% O₂) did not significantly suppress the increase in heart rate in young women (28.1 ± 9.2 years) during treadmill exercise [32]. In contrast, this study found that the HYPER condition suppressed cardiopulmonary stress during maximal exercise, suggesting that the pressurized environment played a role in this effect.

Both W_max_ and W_VT_ during exercise under the HYPO condition were reduced compared to those under the NOR condition, indicating that anaerobic metabolism started at a lower exercise load. As mentioned above, the O_2_ supply to the working muscle was reported to be determined by CaO_2_ × blood flow [26]. In this study, a decrease in SpO_2_ during exercise was observed under the HYPO condition, indicating that CaO_2_ was also reduced under this oxygen condition. The decrease in W_VT_ under the HYPO condition suggested a reduction in the O_2_ supply during exercise regardless of the change in blood flow. Previous studies have reported that exercise under hypobaric hypoxic conditions reduces the O_2_ supply to skeletal working muscles [33–35], which is consistent with our findings. Elevated HR, SBP, and DP throughout exercise under the HYPO condition were estimated as a compensatory response to the reduction in O_2_ supply. In other words, cardiopulmonary stress increased during maximal exercise under the HYPO condition. These findings suggested that exercise under the HYPO condition may be beneficial for athletes and other healthy individuals to improve exercise performance because oxygen deficiency in skeletal muscles could lead to greater tolerance against hypoxia [34].

In clinical practice, exercise is recommended for patients with cardiopulmonary dysfunction to reduce the risk of cardiovascular events [36]. Exercise-induced hypertension is caused by sympathetic overexcitation [37] and is reported to cause organ damage, including left ventricular hypertrophy, atherosclerosis, and renal dysfunction [38]. A previous study reported that the frequency of hypertension during exercise was more observed in patients with cardiopulmonary disease than in healthy individuals [39]. Therefore, exercise load should be restricted to avoid exacerbating their cardiopulmonary disease. This restriction makes it difficult to provide adequate exercise load for patients with cardiopulmonary disease. Our findings suggest that mild hyperbaric hyperoxia could help exercise in patients with cardiopulmonary dysfunction by suppressing hypertension and tachycardia, reducing the risk of cardiovascular complications.

This study had some limitations. First, we did not directly evaluate blood flow during exercise tests. Further studies using near-infrared spectroscopy and doppler ultrasonography are required to clarify this issue. Second, there were variations in participant characteristics. The participants’ ages ranged from twenty-two to sixty years, and their cardiopulmonary functions varied among individuals. However, the wide distribution of these characteristics suggests that the results of this study may apply to other populations. Further clinical studies are needed to investigate the effects of exercise under the HYPER condition on individuals with cardiopulmonary dysfunction. Finally, only a single ramp-exercise protocol was employed in this study. Thus, further investigation is needed on ramp-exercise protocols with varying patterns and intensities.

## Conclusions

Mild hyperbaric hyperoxia (1.3 ATA, 35% O₂) enhanced aerobic metabolism and reduced cardiopulmonary stress during maximal exercise tests. To the best of our knowledge, this is the first study to investigate the clinical benefits of exercise in a mildly hyperbaric hyperoxic environment. Although further clinical research is needed to apply mild hyperbaric hyperoxia in clinical practice, our findings suggested that exercise under a mild hyperbaric hyperoxic environment can be beneficial for individuals with cardiopulmonary dysfunction and may help them exercise safely.

## Supporting information

S1 Dataset All data from the experiment. (XLSX)
